# Supplementary material for: Content-rich biological network constructed by mining PubMed abstracts
Source: BMC Bioinformatics. 2004 Oct 8;5:147. doi: 10.1186/1471-2105-5-147 (PMC528731; doi:10.1186/1471-2105-5-147)
Supplement: Additional File 2 — The original results of the above study (non-essential files are deleted to keep the file size under the limit set by BMC bioinformatics). [file 1471-2105-5-147-S2.bz2 › chilibotAdditionalFile2/dip10/161ID8612709E566/html/ACVR1_ACVR2.html]

 


 **ACVR1** and **ACVR2** 
  
Found 63 abstracts in PubMed, retrieved 10.  
 

 What does Google say? 
 PDF only 
| .edu only 

---

**Interactive relationship** (e.g. stimulation, inhibition, etc)

**Non-interactive relationship** (e.g. studied together, co-existance, homology, etc.)

- these cells were found to express mRNA for BMP receptors including, ActR I  [ **ACVR1** ] , BMPR IA, ActR II  [ **ACVR2** ] , ActR IIB, and BMPR II.  Ref: 11786084 Kidney Int, 2002
- RESULTS Activin A and receptors ActR I  [ **ACVR1** ] , ActR IB, ActR II  [ **ACVR2** ]  are transcribed in corneal fibroblasts.  Ref: 11773015 Invest Ophthalmol Vis Sci, 2002
- Furthermore, transfection with wild type activin receptor  [ **ACVR2** ]  type I  [ **ACVR1** ]  results in activin mediated suppression of cell proliferation.  Ref: 12364468 J Clin Endocrinol Metab, 2002
- A mechanistic explanation for increased pSmad1 5 staining during inflammation was provided by the upregulated expression of all the BMP type I receptors, i.e., activin receptor  [ **ACVR2** ]  like kinase ALK 2  [ **ACVR1** ] , ALK3, and ALK6, in the inflamed bronchial epithelial cells.  Ref: 12151307 Am J Respir Cell Mol Biol, 2002
- Constitutively active forms of BMP type I receptors BMPR IA and BMPR IB and those of activin receptor  [ **ACVR2** ]  like kinase ALK 1 and ALK 2  [ **ACVR1** ]  maintained differentiation of chondrocytes in the presence of retinoic acid.  Ref: 12565908 Biochem Biophys Res Commun, 2003
- Three lysines, Lys 431, Lys 440, and Lys 441 in p50 were all acetylated in vitro, and a sequence similarity among p50, p53, Tat, and activin receptor  [ **ACVR2** ]  type I  [ **ACVR1** ]  on these particular lysines was observed.  Ref: 11739381 J Biol Chem, 2002
- Activin receptor  [ **ACVR2** ]  like kinase ALK 1, ALK 2  [ **ACVR1** ] , ALK 3, and ALK 6 were able to reproduce the hypertrophic maturation of chondrocytes induced by BMP 2.  Ref: 12082094 J Biol Chem, 2002
